# Improving Primary Healthcare Education through lessons from a flock of birds

**DOI:** 10.1038/s41533-023-00355-6

**Published:** 2023-09-30

**Authors:** Ioanna Tsiligianni, Antonios Christodoulakis, Siân Williams

**Affiliations:** 1 Department of Social Medicine, School of Medicine, University of Crete, Heraklion, Crete Greece; 2 International Primary Care Respiratory Group, London, UK

Every year, when the seasons change, flocks of birds migrate (approximately 20% of all species) to find new resources and breed^1,2^. Their journey will overcome multiple challenges (e.g., haematological, metabolic, and hormonal decreases, due to high energy consumption), until they finally and successfully reach their destination^3^. Similarly, primary care professionals strive in their journey to provide the best quality of care to their patients in an ever-changing, complex and challenging healthcare landscape^4,5^. Healthcare education has emerged as a valid method by which primary care professionals can be guided to overcome these challenges^4^. However, despite the assistance of healthcare educators, primary care professionals still experience challenges that may inhibit their ability to provide the best quality of care to their patients^4^. Interestingly, a flock of birds does not have an educator to nurture and guide them. Therefore, how do birds reach their destinations and what could educators learn from birds to help primary healthcare professionals reach their destination of providing high quality care to their patients? The answers may lie in complexity science. Studies about complexity have shown that a few simple principles guide flocks of birds: separate-avoid crowding; align-steer in roughly the same direction as each other, and cohere-steer towards the center of the mass^6,7^. In this way oceans are crossed, avoiding or overcoming multiple challenges along the way. Formations emerge that can cope with their ever-changing environment.

For example, the V-formation reduces energy consumption and improves locomotor performance by maximising upwash capture^1,8,9^. We believe that healthcare educators can take lessons from flocking birds and improve their approach to help primary care professionals/students reach their destination more effectively. The aim of this editorial was to suggest five practical guiding principles that healthcare educators could implement in their practice to promote improved primary care quality. This is particularly valid for adult learners, with prior experience, compared to undergraduate students. It should be noted that the lessons were also partly inspired by the IPCRG’s flagship educational programme “E-Quality”^10^ and could be implemented in classroom and/or clinical educational settings.

## Set the direction: everyone in a classroom/clinical educational setting should know the goal of the educational activity

The first fact is that in the V-formation all birds can see where they are headed. This means that whether the bird is in the first or last place of the formation, it knows the flock’s destination^1^. Possibly, honking plays a role in this as a form of communication between the birds in the formation. Similarly, a healthcare educator could set the direction by describing what good care looks like, and explaining how the teaching goals, course content, and relation/application to primary care for each subject or lesson in any educational activity. Furthermore, healthcare educators could emphasise to their students the importance of sharing knowledge that helps move the flock to the destination.

## Cooperating with the students sustains energy and will help to achieve the teaching goals of each lesson more easily than teaching alone

The second fact is that because it is more energy efficient, the V-formation allows a flock of birds to fly further than flying alone^11^. This occurs because the leading bird creates an updraft with its wings, which the following bird takes advantage of and stays afloat more easily than flying alone^1^. The lesson from this occurrence is that by cooperating with their students and acting as mentors, healthcare educators can more easily achieve their teaching goals. In addition, healthcare educators, by understanding the knowledge and capabilities of each student, could urge more experienced students to help less experienced ones and promote understanding and collaboration between professions.

## Taking turns in the leading position could help reach the teaching goals of each lesson

The third fact is that during flight, birds interchange positions, still steering to the direction that has been set^8^. Interchanging is especially important (i.e., energy efficient) for the lead position because the first bird receives no benefit from the formation and becomes tired more easily^8^. Therefore, by changing their positions, the birds help each other to continue moving forward together and reach their destination. The lesson is that educators should create the environment where students feel able to take the initiative during lessons, thus reconfirming the need for a shift from teacher-centered to learner-centered teaching^12^. Therefore, educators could adopt active learning methods and encourage their students to participate, teach, and share their clinical experiences during lessons. This could be achieved by giving oral presentations and team-building-based tasks that also promote collaboration among students. Consequently, students will have the opportunity to take the lead in a controlled manner during lessons.

## Educators should recognise the educational needs of their students and adapt

The fourth fact is that, during flight, individual birds may face a problem (e.g., tiredness, malnutrition, etc.) and exit the formation without ever returning to it^1^. These birds never reach their destination and may even perish due to lack of resources. Learning from this, healthcare educators should understand that they are facilitators of learning for their students, and need to adjust to their reality on any given day^13^. Furthermore, adult learners tend to learn not according to the discipline of subjects for a possible but distant problem, but rather according to their immediate problem-solving needs^13^. Therefore, to better facilitate the students’ learning, educators should offer them practical knowledge that will help them overcome short-term problems and optimise their thinking process rather than replace it^13^. This means that in a complex world of so much knowledge, educators need to set a direction and create an environment where learners can take what they want and need, and build resourcefulness. In an effort to create such environment, educators should try to incorporate the voices of people with lived experience, their own knowledge/experience, what is available online, and the needs and competences of the learners into their lessons.

## Constantly monitor and evaluate the interactions and make minor adjustments to keep the pace, energy and steerage on course

The fifth fact is that birds must have spatial awareness and the ability to respond when necessary to fly in formation^9^. By observing the flapping motion of nearby flock mates, birds try to place their wings in such a way as to maximize upwash capture. The lesson from this fact is that healthcare educators by monitoring and evaluating the learning environment [meaning: “the social interactions, organisational culture and structures, and physical and virtual spaces that surround and shape the learners’ experiences, perceptions, and pace of learning”^14^], and the behaviors of the learners, could achieve the objectives of each subject/lesson more easily^15^. Furthermore, educators could converse with their students and exchange ideas on how to improve the learning environment and make learning more efficient and engaging^16^.

## Conclusion

In conclusion, this editorial uses the example of flocking and provides five useful principles for healthcare educators to utilise during lessons. We believe that these principles could help healthcare educators make their lessons more efficient, engaging, learner-centered and appropriate for the healthcare challenges faced by the learners. Furthermore, this different approach could help educators guide and nurture primary healthcare professionals to stay on course and reach their destination. Finally, we hope that each time healthcare educators look up at the sky and see birds flying to new horizons, they will reflect on these lessons and improve the quality of their work.
